# Biomass enhancement and activation of transcriptional regulation in sorghum seedling by plasma-activated water

**DOI:** 10.3389/fpls.2024.1488583

**Published:** 2024-11-22

**Authors:** Hong Kwan Beak, Ryza A. Priatama, Sang-Ik Han, Ilchan Song, Soon Ju Park, Young Koung Lee

**Affiliations:** ^1^ Institute of Plasma Technology, Korea Institute of Fusion Energy, Gunsan, Republic of Korea; ^2^ Division of Biological Sciences, Wonkwang University, Iksan, Republic of Korea; ^3^ Upland Crop Breeding Research Division, Department of Southern Area Crop Science, National Institute of Crop Science, Rural Development Administration, Miryang, Republic of Korea; ^4^ Division of Applied Life Science and Plant Molecular Biology and Biotechnology Research Center (PMBBRC), Gyeongsang National University, Jinju, Republic of Korea; ^5^ Plasma and Nuclear Fusion, University of Science and Technology, Daejeon, Republic of Korea

**Keywords:** plasma-activated water, sorghum seedling, biomass enhancement, transcriptional regulation, structural changes

## Abstract

**Introduction:**

Recent advancements in agricultural technology have highlighted the potential of eco-friendly innovations, such as plasma-activated water (PAW), for enhancing seed germination, growth, and biomass production.

**Methods:**

In this study, we investigated the effects of PAW irrigation on young sorghum seedlings through phenotypic and transcriptional analyses. We measured growth parameters, including seedling height, stem thickness, and biomass, across five sorghum varieties: BTx623, Sodamchal, Noeulchal, Baremae, and Hichal. Additionally, we performed detailed analyses of stem cross-sections to evaluate the structural changes induced by PAW. Whole transcriptome analysis was conducted to identify differentially expressed genes (DEGs) and to perform Gene Ontology (GO) analysis.

**Results:**

Phenotypic analysis revealed significant growth enhancements in PAW-treated seedlings compared to the control group, with notable increases in seedling height, stem thickness, and biomass. Stem cross-section analysis confirmed that PAW treatment led to the enlargement of primordia tissue, leaf sheath (LS1 and LS2), and overall stem tissue area. Transcriptomic analysis revealed that 78% of the DEGs were upregulated in response to PAW, indicating that PAW acts as a positive regulator of gene expression. Gene Ontology (GO) analysis further showed that PAW treatment predominantly upregulated genes associated with transmembrane transport, response to light stimulus, oxidoreductase activity, and transcriptional regulation. Additionally, an enriched AP2/EREBP transcription binding motif was identified.

**Conclusion:**

These findings suggest that PAW not only enhances sorghum seedling growth through transcriptional regulation but also has the potential to optimize agricultural practices by increasing crop yield. The upregulation of genes involved in critical biological processes underscores the need for further exploration of PAW’s potential in improving the productivity of sorghum and possibly other crops.

## 1 Introduction

Food shortages due to increasing population, climate change, and deteriorating environmental conditions have become a major issue that requires attention and immediate solutions (Anderson et al., 2020; Duchenne-Moutien and Neetoo, 2021). Traditionally, various strategies have been employed to improve agricultural production through physical and chemical methods, such as seed priming, stratification, scarification, disinfectants, fungicides, hormones, and fertilizers (Araújo et al., 2016; Kiiski et al., 2016; Pawar and Laware, 2018). However, these methods often require expensive equipment, are time-consuming, and are sometimes impractical. Furthermore, modern agriculture relies heavily on chemical fertilizers, pesticides, and other chemicals to ensure healthy growth and higher yields; however, these approaches lead to environmental pollution and health risks (Savci, 2012; Sun et al., 2018; Bijay-Singh and Craswell, 2021).

Emerging sustainable technologies, such as atmospheric cold plasma (CAPP), ion beams, and nanoparticles, are becoming increasingly popular in agriculture because of their advantages in seed sterilization, disease control, and enhancement of seed germination, seedling growth, and plant resistance to stress (Bourke et al., 2018; Usman et al., 2020; Mohammadi et al., 2021). Among these, CAPP technology has shown the potential to stimulate germination, improve cultivation and growth, and enhance secondary metabolite production (Thirumdas and Sarangapani, 2015; Gao et al., 2022; Li et al., 2022a).

The application of CAPP technology to improve crop yield contributes to plant growth during plant development, ultimately increasing plant yield, mainly reactive oxygen and nitrogen species (RONS). Plasma discharge produces high-energy electrons that collide with water and cause various reactions, including ionization and dissociation (Małajowicz et al., 2022). This phenomenon generates several reactive species. Reactive species are further classified as reactive oxygen species (ROS), hydrogen peroxide (H_2_O_2_), ozone (O_3_), superoxide (O_2_^-^), reactive nitrogen species (RNS), nitric oxide radicals (•NO), nitrogen dioxide radicals (•NO_2_), peroxynitrite (ONOO^-^), nitrite (NO_2_^-^) and nitrate (NO_3_^-^). The generation of RONS from plasma discharge is considered the main factor that enhances crop growth and sterilization, and nitrate in RNS is a stable ion and the main source of nitrogen, which is a macronutrient for plant growth and development (Vidal and Gutiérrez, 2008; Vidal et al., 2015). It also affects seed germination, root and leaf growth, root structure, flowering time, branching, plant aging, and yield (Kant et al., 2008; Wang et al., 2021). Recent studies have shown that plasma treatment improves the yield and biomass of crops, including horticultural crops, mainly because of the nitrate content in the plasma-activated water (PAW) (Lamichhane et al., 2021; Lukacova et al., 2021).

Plasma treatments can be broadly categorized into direct and indirect applications, each having distinct impacts on plant growth and development. In direct plasma treatment, the plasma directly interacts with the target biological organisms, where charged particles and reactive species, including ozone, hydrogen peroxide, and OH radicals, immediately affect the biosamples. For instance, using a dielectric barrier discharge (DBD) device at 87 W power for 10 min promotes the growth of pea seeds, whereas treatment at 60, 80, and 100 W for 15 s enhances the vitality of tomato germination and growth (Jiang et al., 2018; Gao et al., 2019).

In contrast, indirect plasma treatments do not directly expose the samples to plasma. Instead, plasma-activated species in the gas phase or PAW are applied to the samples. These treatments activate signaling pathways that promote vegetative and root growth, seed germination, and plant reproduction without direct plasma exposure. For example, PAW produced using DBD with a gas mixture of Ar, Nr, and On has been shown to enhance seed germination and seedling growth in lettuce (*Lactuca sativa* L.) with a treatment of 24 kV for durations ranging from 5 to 20 min (Than et al., 2022). Similarly, plasma jet-treated water used for irrigation, with plasma treatment times of 15 and 30 min, has improved tomato seedling growth (Adhikari et al., 2019).

Moreover, combining direct and indirect plasma treatments can further enhance plant growth and seed germination. Studies using plate-to-plate double DBD for seed treatment and cylindrical double DBD for PAW irrigation have demonstrated significant growth promotion in crops such as radishes, tomatoes, and peppers (Sivachandiran and Khacef, 2017). Additionally, low-pressure glow air discharge at 100 Torr and 9 W for seed treatment, combined with PAW foliar application using a plasma jet at 16 W, has been shown to increase plant growth in rice (Rashid et al., 2021). These findings indicate that cold plasma technology is a versatile tool for enhancing plant development and seed germination. However, the effectiveness of plasma treatments is highly dependent on the specific conditions tailored to each plant species, suggesting that customized treatment protocols are necessary to optimize outcomes (Lyu et al., 2023).

The utilization of CAPP in agriculture requires an understanding of the fundamental mechanisms underlying the phenomenon of plasma in crops that promote plant growth and confirm the efficiency of plasma treatment (Priatama et al., 2022). Recent studies have investigated the molecular mechanisms, such as gene expression from single genes to whole transcriptome analysis, protein expression analysis, and epigenetic regulation, that underlie the effects of plasma on the regulation of seed germination, plant growth, and biomass (Adhikari et al., 2019; Javed et al., 2023; Gupta et al., 2024; Kaushik et al., 2024). Cell-level observations were performed after plasma treatment in addition to the molecular level. In *Arabidopsis*, tobacco, and peanuts, plant growth is promoted after exposure to PAW because of changes in cell size and number, which are key factors in determining tissue and plant size (Lee et al., 2020; Ka et al., 2021; Song et al., 2023). PAW irrigation has been shown to improve the expression of growth and defense response genes in tomatoes, and plasma treatment of *Arabidopsis* seeds promotes the expression of specific genes related to GSH metabolism, mitogen-activated protein kinase signaling, and plant resistance to pathogens (Adhikari et al., 2019; Cui et al., 2021). Recently, proteome and metabolome studies have demonstrated that PAW treatment of radish improves the accumulation of functional compounds (Gupta et al., 2024), and proteomic studies have examined the plasma treatment of African marigold seeds to generate differential 491 protein group, which are involved in ROS homeostasis, green plant photosynthesis, and energy metabolism (Javed et al., 2023). However, the molecular mechanisms underlying plasma-induced growth and biomass increment are largely undiscovered, particularly in crops with significant agricultural value and responsiveness to PAW, depending on crops according to the plasma device.

To understand the phenotypic sensitivity, modification at the cellular level, and molecular factors after PAW treatment among sorghum varieties, we performed phenotypic examination and transcriptome analysis of the stem tissue. Surface dielectric barrier discharge (sDBD) was used as a plasma-generating device to produce PAW dissolved in ionized gas in distilled water (DW). We found remarkable differences in biomass increments in sorghum seedlings in response to PAW treatment due to activated transcriptional regulation.

## 2 Materials and methods

### 2.1 Experimental setup for plasma device and PAW production

**Figure 1:**
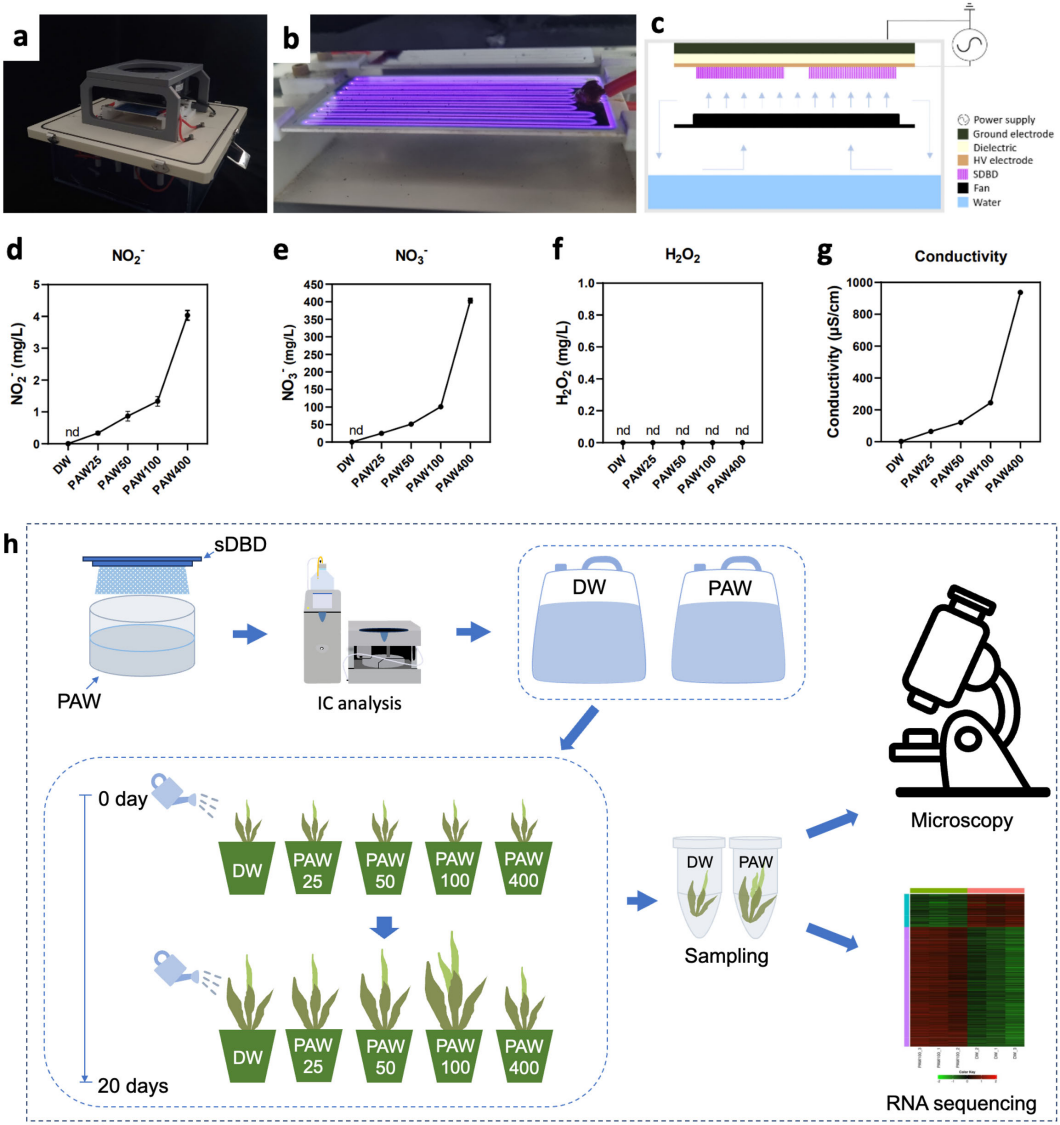
Plasma-generated device and overview of experimental design used for sorghum seedling growth. **(A–C)** Settings for producing PAW: **(A)** sDBD device **(B)** sDBD electrodes **(C)** Schematic diagram of sDBD device used for PAW generation. **(D–G)** Physicochemical properties of PAW: **(D)** NO_2_^-^ concentration, **(E)** NO_3_^-^ concentration, **(F)** Hydrogen peroxide (H_2_O_2_), and **(G)** conductivity. **(H)** Schematic diagram of plant growth under PAW irrigation during the developmental stage.

The PAW was generated using sDBD in gas-tight containers (**Figure 1A**), as previously described (Lee et al., 2020; Ka et al., 2021). The electrodes used consisted of stainless steel (power supply and ground) with an aluminum oxide plate (1 mm thick) between the electrodes. The sDBD reactor used in the experiment has two electrodes at the top, and its input power and driving frequency are 10 W and 17 kHz, respectively. Commercial fans (15-LED 120, Aone, China) were used to dissolve the plasma gas in water. Two liters of deionized water (DW) were treated with sDBD to obtain PAW25 (6 min), PAW50 (12 min), PAW100 (25 min), and PAW400 (90 min). We conducted the experiment under five treatment conditions (DW, PAW25, PAW50, PAW100, and PAW400). The nitrate content in PAW was directly proportional to the plasma exposure times. PAW25, PAW50, PAW100, and PAW400 correspond to exposure times of 6, 12, 25, and 90 minutes, respectively, as shown in **Figure 1D**. This correlation is based on our previous studies (Lee et al., 2020; Ka et al., 2021), where we systematically analyzed the impact of varying plasma exposure times on the chemical composition of PAW.

### 2.2 Physicochemical analysis of PAW

The chemical characteristics of PAW were analyzed by measuring the anion content using ion chromatography (ICS-2100, Thermo Dionex, Sunnyvale, CA, USA), which was conducted one day after generating PAW. To quantify the anions, a four-point method was used to measure the standard sample (Dionex Seven Anion Standard II [in DW]) at concentrations of 5, 10, 50, and 100 mg/L, and the pH and conductivity were determined using an Orion Versa Star Pro (Thermo Scientific, Waltham, MA, USA). The pH was adjusted to 5.6-5.8 using 0.1 N and 1 N KOH solution to ensure it is suitable for plant growth. Hydrogen peroxide (H_2_O_2_) was measured using a Humas HS-H_2_ O_2_ -L kit (Humas, Daejeon, Republic of Korea). All measurements were performed in triplicates.

### 2.3 Plant materials and growth conditions

In this study, we used the sorghum varieties Sodamchal, Noeulchal, Baremae, and Hichal, obtained from propagation in the field (National Institute of Crop Science, Miryang, Republic of Korea). Seed propagation and all experiments were performed following common agricultural practices and complied with relevant institutional regulations. The experiment was conducted in a growth chamber (GC-S, Jeiotech, Seoul, Rep. of Korea) in light 16 h and dark 8 h conditions with 26 °C at 60-70% humidity. For seedling phenotypes, sorghum seeds were germinated on filter paper under the appropriate treatment conditions (DW, PAW). Seedling phenotypes were measured on the 20th day after sowing.

For the phenotypic comparison, seeds were germinated by laying paper on a plate and adding 5 mL of each solution for each condition; on the 2nd day after germination, the seeds were transplanted into the soil and harvested by growth for 20 days. Throughout the experiment, day and night temperatures and relative humidity were maintained at 26°C and 60%, respectively. Sorghum plants were regularly irrigated with PAW at all growth stages to ensure consistent treatment.

### 2.4 Leaf phenotype and chlorophyll content measurement

Sorghum leaves were collected from the fourth leaf (or the third leaf if the fourth was less developed) of the plants 20 days after sowing. The leaves were fixed using A4 double-sided tape and scanned with a scanner, and the leaf characteristics (leaf area, length, and width) were measured using ImageJ software version 1.54k (https://imagej.net/ij/).

To measure total chlorophyll content in leaves, the final fully expanded leaves were cut, weighted and ground in tissue homogenizer. The chlorophyll was extracted from leaf by 80% acetone, incubated on dark and repeated until all chlorophyll was dissolved. Total chlorophyll content was measured using a spectrophotometer (Hach DR6000, Loveland, CO, United States), with absorbance readings taken at A665 and A648. Biological replicates consisted of at least five individual plants for each treatment condition (DW, PAW25, PAW50, PAW100, PAW400).

### 2.5 Microscopy observations

After cutting the stem–root interface of the sorghum seedlings, a portion approximately 4 cm above the bottom of the stem (meristem location) was cut using a razor blade. It was directly stained with 0.05% toluidine blue O for approximately 1 min and then washed 3-4 times with DW. Samples were observed under a Nikon SMZ18 optical microscope (Nikon, Tokyo, Japan) using imaging software (NIS-Elements, Nikon, http://www.microscope.healthcare.nikon.com/). Size and area measurements of the cut biological material were performed using the ImageJ software.

### 2.6 Total RNA extraction and library preparation

Total RNA was isolated from the shoot apical region of sorghum, specifically from a segment spanning 0.5 cm to 1 cm from the root-shoot junction from 20 days seedlings. The isolation was performed on seedlings cultivated under both DW and PAW100 conditions. Fresh seedlings were immediately frozen and pulverized in liquid nitrogen. Subsequently, RNA was extracted using the TRIzol Plant RNA Extraction Kit, following the manufacturer’s standard protocol.

Library preparation was initiated for RNA sequencing by assessing the quality and quantity of the extracted RNA using Bioanalyzer 2100. High-quality RNA was used to construct sequencing libraries. This process involves RNA fragmentation, synthesis of the first and second cDNA strands, end repair, A-tailing, adapter ligation, and enrichment of the library through PCR. The prepared libraries were quantified and validated for quality before sequencing on an Illumina high-throughput sequencing platform. The RNA-seq reads were deposited in the NCBI Sequence Read Archive under BioProject accession PRJNA1173527.

### 2.7 RNA sequencing analysis and differential expression

RNA sequencing was performed to investigate gene expression patterns and identify differentially expressed genes (DEGs) under specific experimental conditions. Raw RNA-Seq reads were aligned to the Sorghum bicolor reference genome, specifically version NCBIv3, using Kallisto, a pseudo-alignment-based tool that efficiently estimates abundance levels and provides counts per million (CPM) and transcripts per million (TPM) values (Bray et al., 2016). The reference sequence, designated as *Sorghum_bicolor*, Sorghum_bicolor_NCBIv3.dna.toplevel.fa, and the corresponding annotation files Sorghum_bicolor.Sorghum_bicolor_NCBIv3.57. gff3 were accessed and downloaded from the Ensembl Plant database (Cunningham et al., 2022).

Differential expression analysis was subsequently conducted using iDEP version 1.13, installed locally, with Ensembl Release 107 integrated for genomic data annotation (Ge et al., 2018). The iDEP platform facilitated the efficient handling of data preparation, normalization, and statistical analysis using the DEseq2 1.44.0 package in R 4.4.0 (Love et al., 2014). DEGs were defined based on criteria including a 1.5-fold change and a false discovery rate (FDR) threshold of < 0.05. Hierarchical clustering was used to analyze the gene expression patterns, as assessed by log_2_TPM, across both groups.

### 2.8 GO analysis

GO analysis was also performed using iDEP, which incorporates ShinyGO (version 0.77) for functional enrichment analysis (Ge et al., 2020). This analysis included both enrichment and GO network analyses, which enabled the identification and visualization of enriched GO terms across biological processes (BPs), cellular components (CCs), and molecular functions (MFs). After identifying the significant GO categories, we isolated and extracted expression values (TPM) using the dplyr package (version 1.1.4). These values were then used to generate heatmaps. Heatmap analysis of the gene expression patterns was performed using the pheatmap package (version 1.0.12) in R in the RStudio environment (version 2024.04.0, Build 735).

### 2.9 Promoter motif analysis

To identify conserved motifs among the promoter sequences of highly enriched genes, we collected 2000 base pair sequences upstream of the transcription start site from 285 genes associated with significantly enriched GO categories across BPs, CCs, and MFs. The MEME Suite (version 5.5.5) was used for motif discovery (Bailey et al., 2015). The analysis was performed with the following parameters: the top six motifs were displayed, with a motif length of 20, and all other settings were set to default. For visualization, the identified motifs were further analyzed using TBtools [https://github.com/CJ-Chen/TBtools] (Chen et al., 2020) (Chen et al., 2020). Subsequently, the conserved motifs were subjected to a motif comparison analysis using TomTom (version 5.5.5), a motif comparison tool that aligns and compares discovered motifs against known motif databases. The comparisons were based on the *Arabidopsis thaliana* DNA Affinity Purification Sequencing (DAP) study, as reported by O’Malley et al. (2016).

### 2.10 Statistical analyses

GraphPad Prism 9.0 (GraphPad Software, Inc., San Diego, CA, United States) were used for the statistical analysis. Unless described in the figure legends the statistical analysis and comparison among samples were made using one-way ANOVA followed by Tukey’s HSD correction for multiple comparisons. If the *P-*value was less than 0.05, the different uppercase letters denoted significant differences among samples. The data presented as mean ± SD.

## 3 Results

### 3.1 Preparation of PAW

To understand the effects of PAW on early seedlings of the sorghum variety, PAW was produced using an sDBD device with various plasma exposure times (**Figures 1A–C**). The physical and chemical properties of PAW change depending on the plasma treatment time of the sDBD device (Ka et al., 2021). Using sDBD, we previously reported that the main molecular spectra, including the nitrogen primary negative system (N_2_ FNS) and nitrogen secondary positive system (N_2_ SPS) in the ranges of 280-296 and 390-440 nm, were detected by optical emission spectroscopy (OES) analysis during air discharge (Ka et al., 2021). The standardization of PAW25, PAW50, PAW100, and PAW400 were designated based on their nitrate contents, which were 25, 50, 100, and 400 correspond to exposure times of 6, 12, 25, and 90 minutes, respectively. In detail, the NO_2_ **^-^** and NO_3_ **^-^** contents increased proportionately with the duration of plasma exposure time (**Figures 1D, E**, **Supplementary Figure 1**), whereas H_2_O_2_ was hardly detected regardless of the PAW exposure time (**Figure 1F**). Similar to the nitrite and nitrate contents by PAW concentration, the conductivity was found to increase proportionally with increasing nitrate concentration (**Figure 1G**). **Figure 1H** shows a schematic diagram of the experimental process. First, PAW was prepared using sDBD on DW by plasma treatment, and IC analysis was performed to confirm the PAW content. After confirming the presence of DW and PAW, irrigated sorghum was grown, and 20-day-old plants were sampled and subjected to microscopic analysis and RNA sequencing.

### 3.2 PAW effect on sorghum varieties

To evaluate the effect of PAW on sorghum at the seedling stage, various PAW concentrations were tested in a preliminary experiment, including PAW25, PAW50, PAW100, PAW200, PAW300, and PAW400. The results showed that biomass consistently increased with PAW25, PAW50, and PAW100. However, PAW200 and PAW300 resulted in similar or slightly decreased growth compared to PAW100, although both were still greater than DW. A decline in growth relative to DW was observed at PAW400. Based on these findings, we selected PAW25, PAW50, and PAW100 as concentrations that enhance biomass, whereas PAW400 was chosen for its inhibitory effect (**Supplementary Figure 2**).

**Figure 2:**
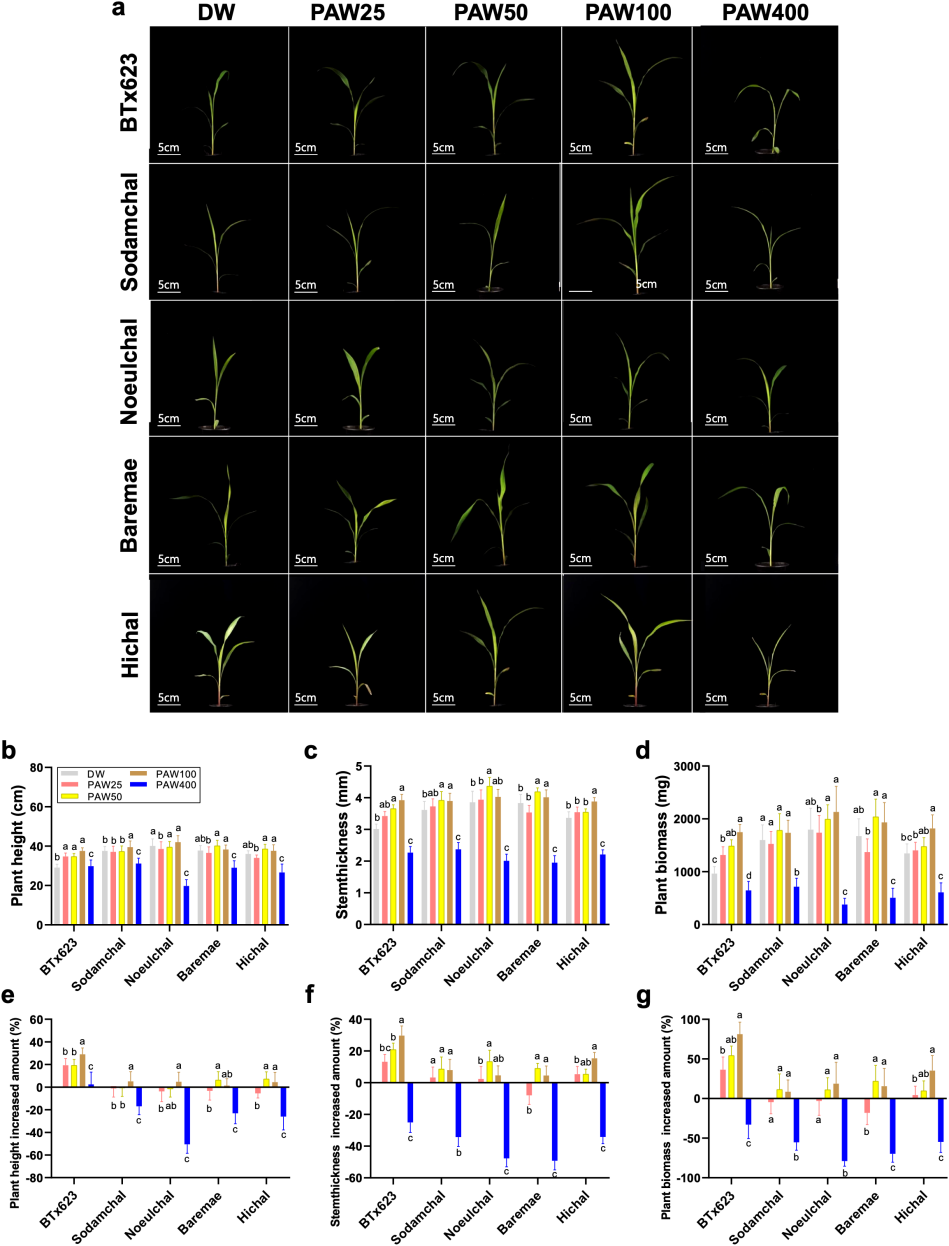
PAW increases growth in the early developmental stages of sorghum varieties. **(A)** Seedling phenotypes in PAW treatments at different concentrations compared to DW. Scale bar: 5 cm. **(B–D)** Quantification of seedling phenotype and biomass **(B)** plant height, **(C)** stem thickness, **(D)** plant biomass from 25 plants at the seedling stage. **(E–G)** Comparison of increase in phenotypic data **(E)** plant height, **(F)** stem thickness, and **(G)** biomass (DW vs. PAW). Bar plots represent mean ± standard deviation (SD), and values are from at least three replicates for each, and different letters indicate statistically significant differences (ANOVA, Tukey’s HSD, *P* ≤ 0.05). Seedlings were grown in soil for 20 days. All phenotypes were recorded 20 days after sowing.

To further examine the effect of PAW on different sorghum varieties, we selected five varieties: BTx623, Sodamchal, Noeulchal, Baremae, and Hichal. Plant height, stem thickness, and biomass were measured to assess their sensitivity to PAW treatment (**Figures 2A–D**). Additionally, we analyzed the increase in these phenotypic traits following PAW irrigation compared to DW (**Figures 2E–G**). After 20 days of PAW irrigation, the sorghum varieties exhibited differential growth responses at PAW25, PAW50, PAW100, and PAW400 concentrations (**Figure 2**).

In terms of plant height, BTx623 showed a proportional increase with PAW25, PAW50, and PAW100, correlating with nitrate concentration. However, the Sodamchal, Baremae, and Hichal varieties showed no response at PAW25, the lowest nitrate condition. In contrast, plant height was significantly inhibited in all varieties under PAW400 (**Figures 2A, B**), with PAW100 producing the tallest plants among the five varieties (**Figures 2A, B**). In addition, stem thickness in BTx623 increased even at the lowest concentration of nitrate, and progressively increased to PAW100, depending on the nitrate concentration. Interestingly, PAW100 exhibited the highest stem thickness across most varieties, whereas PAW400 exhibited the lowest stem growth across all varieties (**Figures 2C, F**).

All sorghum varieties gradually responded to biomass under low nitrate conditions in PAW25 and PAW50 and had a robust reaction in PAW100 (**Figures 2D, G**). They decreased the biomass in PAW400, which had the highest nitration water content. Additionally, leaf phenotypes (leaf area, leaf length, leaf width, and fully expanded leaf number) and chlorophyll content were measured in response to PAW concentrations across all sorghum cultivars (**Supplementary Figure 3**). In all cultivars, leaf area gradually increased from PAW25 to PAW100, and in four cultivars, except Noeulchal, PAW400 showed a decreased leaf area compared to DW (**Supplementary Figure 3A**). Leaf length slightly increased to nitrate concentration from PAW25 to PAW100, similar to leaf area (**Supplementary Figure 3B**). Leaf width increased gradually from PAW25 to PAW100 in BTx623 and Noeulchal (**Supplementary Figure 3C**). The number of fully expanded leaves followed a similar pattern across all cultivars, with the highest values ​​at PAW50 and PAW100 and the lowest values ​​at DW and PAW400 (**Supplementary Figure 3D**). Chlorophyll content varied across cultivars and nitrate concentrations, and no significant correlation was observed (**Supplementary Figure 3E**). The PAW 100 treatment of young sorghum seedlings served as the optimal PAW condition for the highest plant height, stem thickness, and biomass, indicating that sorghum plants exhibit distinct physiological responses when exposed to varying amounts of PAW. Among the sorghum varieties, the strongest response to PAW in terms of plant biomass was observed in BTx623, whereas the other varieties showed weak phenotypic sensitivity. These results imply that PAW treatment regulates the growth of the BTx623, Sodamchal, Noeulchal, Baremae, and Hichal sorghum varieties, which exhibit variable phenotypic sensitivities to PAW exposure time.

### 3.3 Sorghum stem cross-section phenotype and area calculation

**Figure 3:**
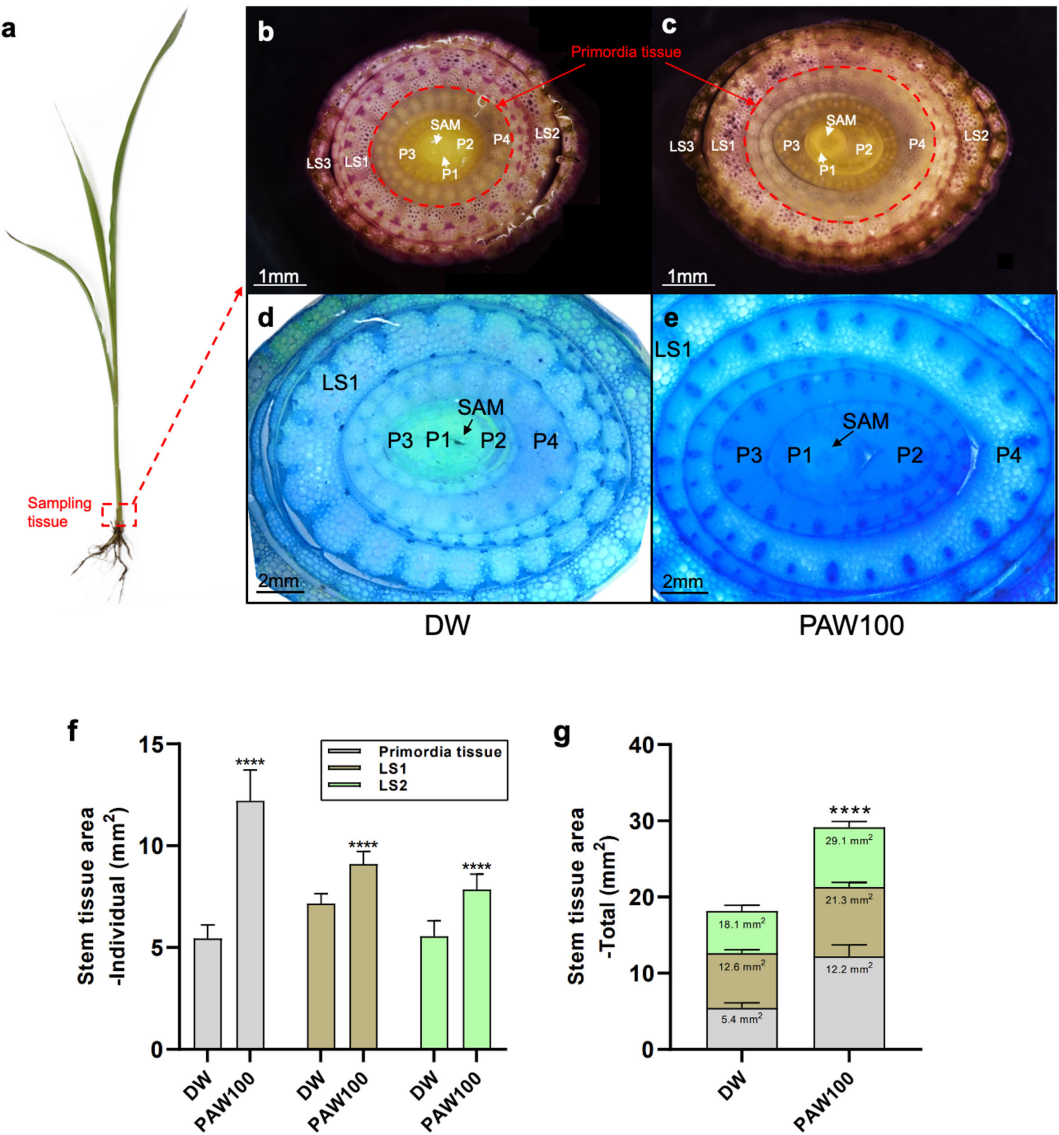
Comparison of stem cross sections of seedlings grown under PAW treatment conditions. **(A)** BTx623 seedling phenotype at 20 days in sorghum. Red box: section tissue. **(A, B)** Phenotypic comparison of DW and PAW100 stem sections. **(D, E)** Comparison of phenotypes in primordia tissue stained with toluidine blue (TBO, blue). Left: treatment DW, right: treatment PAW100. SAM: shoot apical meristem, P: leaf primordia, LS: leaf sheath, red circle: primordia tissue. Photos were taken 20 days after sowing. Scale bar: **(B, C)** 1 mm and **(D, E)** 2 mm. **(F, G)** Quantification of stem tissue area at 20 days after sowing. Bar plots represent mean ± standard deviation (SD), and values are from at least three replicates for each (Student’s t-test, ****P < 0.0001). All phenotypes were recorded 20 days after sowing.

The effect of PAW treatment on specific sorghum tissues was investigated at the cellular level. Phenotypic experimental results from sorghum varieties confirmed the effect of PAW on plant height and stem thickness, resulting in increased biomass in sorghum. We chose stem tissue to further examine the effect of PAW treatment on sorghum tissue and its transcriptome profile because of the strong stem thickness phenotype. Stem tissues included shoot apical meristem (SAM) tissues sampled from the DW and PAW100 treatment groups (**Figure 3A**). Under DW conditions, the three forms of dissected tissues were the leaf sheath (LS), leaf primordia (P), and SAM; the P and SAM tissues were identified as primordial tissues (**Figure 3B**). In the case of DW, the location of the tissue type was organized in the order of SAM, P 1-3, and LS 1-3 from inside to outside. There were no significant differences in the order of tissues or anatomical differences between DW and PAW100. However, the PAW100 condition had an overall larger tissue area, and the primordia tissue, including SAM, P1, P2, P3, and P4, of stem tissue clearly validated that PAW100 had substantially larger stem tissue than DW (**Figures 3B–E**). Specifically, the combination of all tissue areas, primordia tissue, LS1, and LS2 areas was significantly increased in PAW100 condition, 124% in primordia tissue, 27% in LS1, and 42% increment in LS2, respectively (**Figure 3F**). As a result, the overall stem tissue area was enhanced, and sorghum primordia were the most responsive stem tissues to PAW100 treatment. (**Figure 3D**).

### 3.4 RNA sequencing analysis in DW and PAW100

**Figure 4:**
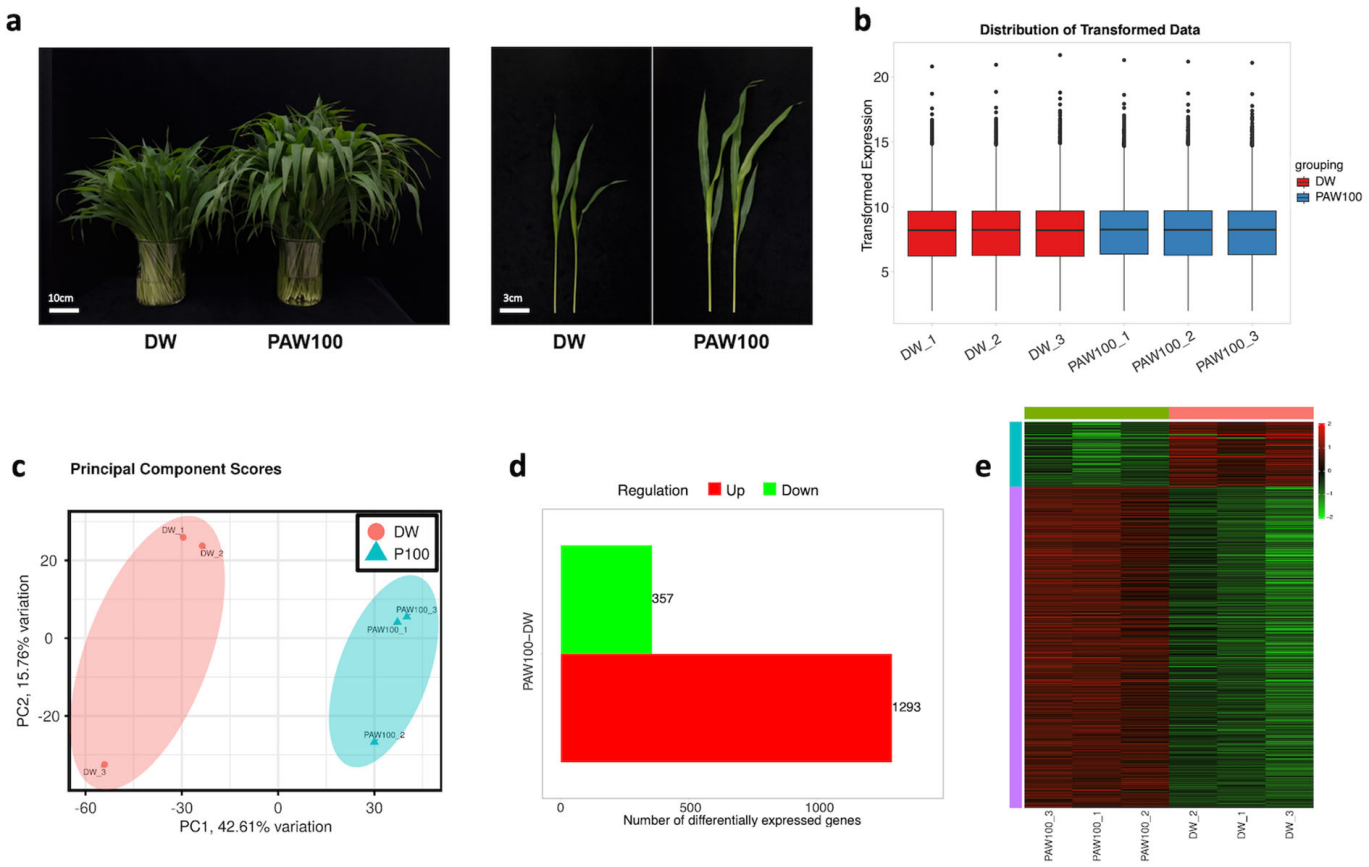
RNA-seq analysis from sorghum seedling stem tissue treated with PAW. **(A)** Seedling phenotypes used for RNA sequencing. Left: total seedling phenotypes (scale bar: 10 cm), right: individual seedling phenotypes (scale bar: 3 cm). Photos were taken 20 days after sowing. **(B)** Distribution of transformed expression in all RNA-seq samples. **(C)** Principal component analysis individual replicates among samples. **(D)** Identified differentially expressed genes (DEGs) in transcriptome of DW vs PAW100. **(E)** Hierarchical clustering and heat map of differentially expressed genes based on the expression levels (TPM). Heatmap from raw TPM and converted to Z-scores.

To understand the transcriptional regulation underlying plasma-induced sorghum growth enhancement, RNA sequencing was performed on six samples of BTx623 treated with DW and PAW100. As shown in **Figures 2**, **3**, the responsiveness to PAW between DW and PAW100 was distinct in the stems of the BTx623 inbred variety, which served as the current reference genome for sorghum (**Figures 2**–**4A**). Total reads mapped to reference with mapping rate at 89-93% (**Supplementary Table 1**). The total raw read counts for each library of stem samples in DW and PAW100 are displayed in a bar graph, with slight variations in library size (**Supplementary Figure 4A**). To effectively control the mean-dependent variance, we performed a normalized log (rlog) transformation using the DESeq2 package (**Figure 4B**). Principal component analysis (PCA) was performed on the samples to evaluate the sample variability. Principal component (PC1) explained 42.61% of the variance, and the second principal component (PC2) accounted for 15.76%, effectively distinguishing differences between the treatment groups. PCA demonstrated close clustering of the individual samples within triplicates and a distinct separation between DW and PAW100 treatments along two principal axes (**Figure 4C**), indicating repeatability of the RNA-seq data. The 35,567 sorghum genes mapped to the RNA-seq library were normalized using TPM (transcripts per kilobase million). Selection criteria were applied, requiring at least a 1.5-fold change, a *P*-value of less than 0.05, and a false discovery rate (FDR) < 0.1. Identified 1,650 DEGs between the DW and PAW100 treatment groups, with 1,293 upregulated genes (78.3%) and 357 (21.6%) downregulated genes in PAW100 compared to DW was depicted in volcano plots of **Supplementary Figure 4B** and the number of DEGs in **Figure 4D**. The resulting heatmap, shown in **Figure 4E**, demonstrates the clustering of the samples from individual replicates by the PAW treatment, indicating consistency across replicates and reflecting the response to the PAW100 treatment.

### 3.5 GO analysis

**Figure 5:**
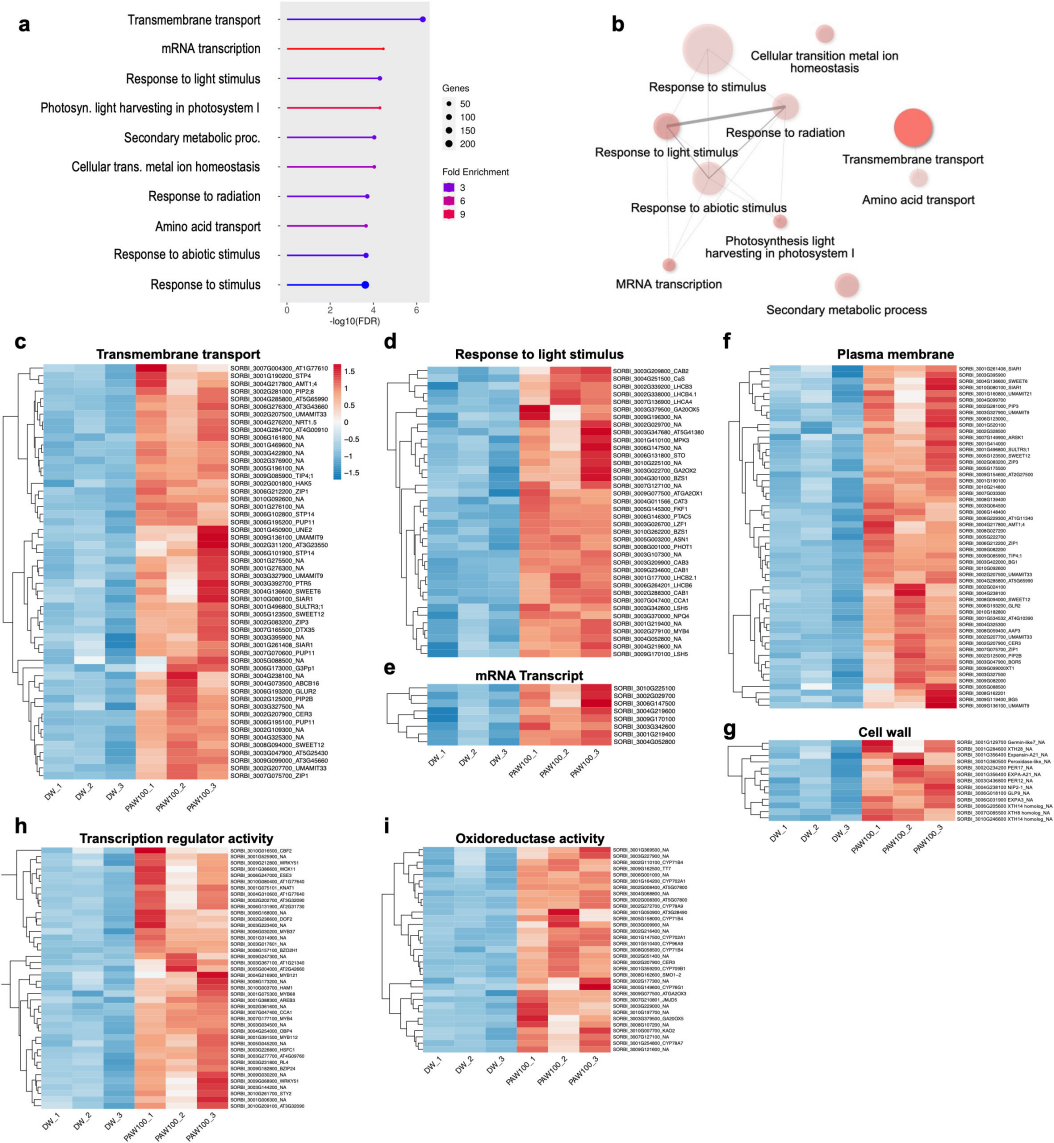
Gene ontology analysis of PAW transcriptome. **(A)** Bar plot of GO Biological Processes. **(B)** Biological Processes GO-network. The size of the dots represents the number of genes associated with each GO term, whereas the color gradient indicates the fold enrichment; a more saturated colors represent the most highly enriched categories. The thickness and shade of the lines denote the strength of interactions between GO terms. **(C–G)** Heatmap representation of gene expression profiles from the PAW treatment transcriptome, categorized by enriched Gene Ontology (GO) terms: **(C–E)** Biological Processes “Transmembrane transport” and “Response to light stimulus;” **(F, G)** shows Cellular Components “Plasma membrane” and “Cell wall;” whereas **(H, I)** represent Molecular Functions “Transcription regulator activity” and “Oxidoreductase activity.” Each panel depicts genes that are highly enriched and upregulated in response to PAW treatment, with color gradients indicating expression levels across different treatment. .

Following the identification of DEGs between DW and PAW100 treatments, GO analysis was performed to elucidate the biological implications of the DEGs (**Supplementary Figure 5**). The analysis revealed significant enrichment of the top 10 GO terms for BPs, CCs, and MFs (**Figure 5A**, **Supplementary Figure 5**). Furthermore, in BPs, transmembrane transport, mRNA transcription, and response to light stimulus were highly enriched (**Figure 5A**); CCs were mainly cell periphery and plasma membrane-related GO was enriched (**Supplementary Figure 6A**); and in MFs, several groups of oxidoreductase activities such as tetrapyrrole binding, monooxygenase activity, heme binding, and iron ion binding were highly enriched. In addition, the DNA-binding transcription factor and transmembrane transporter activity groups were enriched (**Supplementary Figure 6B**).

Additionally, we performed GO network analysis to gain further insight into the overlapping functional relationships and interactions among the enriched gene sets. The enriched network module in the response to the stimulus group was interconnected with other GO terms including responses to radiation, light stimulus, to abiotic stimulus, mRNA transcription, and photosynthesis light harvesting in photosystem I (**Figure 5B**). Similarly, these networks were also detected in CCs and MFs categories, such as the network of cell periphery involved GO module in CCs, and oxidoreductase activity involved module in MFs (**Supplementary Figures 6C, D**). These networks highlight the interplay between different GO terms within the same category of BPs, CCs, and MFs.

Because the upregulation pattern was prominent, we isolated the upregulated genes that were enriched by PAW treatment, especially where the GO-network module showed high interaction among GO-term. As shown in **Figure 5C**, genes involved in transmembrane transport were strongly upregulated, suggesting an enhanced capability for nutrient and ion transport across cellular membranes, which is a key factor for optimal plant growth and function. Furthermore, genes associated with response to light stimuli showed increased expression, which is likely to facilitate adaptations in photosynthetic efficiency and light absorption, which are essential for energy management and biomass production (**Figure 5D**). In addition, the mRNA transcript levels were enhanced by PAW treatment (**Figure 5E**). In the CCs, the expression levels of genes related to the plasma membrane and cell periphery were significantly increased, indicating cellular modifications aimed at increasing structural integrity and biomass accumulation (**Figures 5F, G**). Moreover, in the GO MFs category, upregulation observed in the transcription regulator activity category underscored the activation of critical gene expression regulatory mechanisms that manage stress responses and developmental processes (**Figure 5H**). Similarly, genes involved in oxidoreductase activity, which is crucial for the oxidative stress response and maintenance of redox homeostasis, also showed marked upregulation, enhancing plant resilience against environmental stressors (**Figure 5I**).

### 3.6 Binding motif analysis of enriched GO categories and Transcription Factor (TF) expression

**Figure 6:**
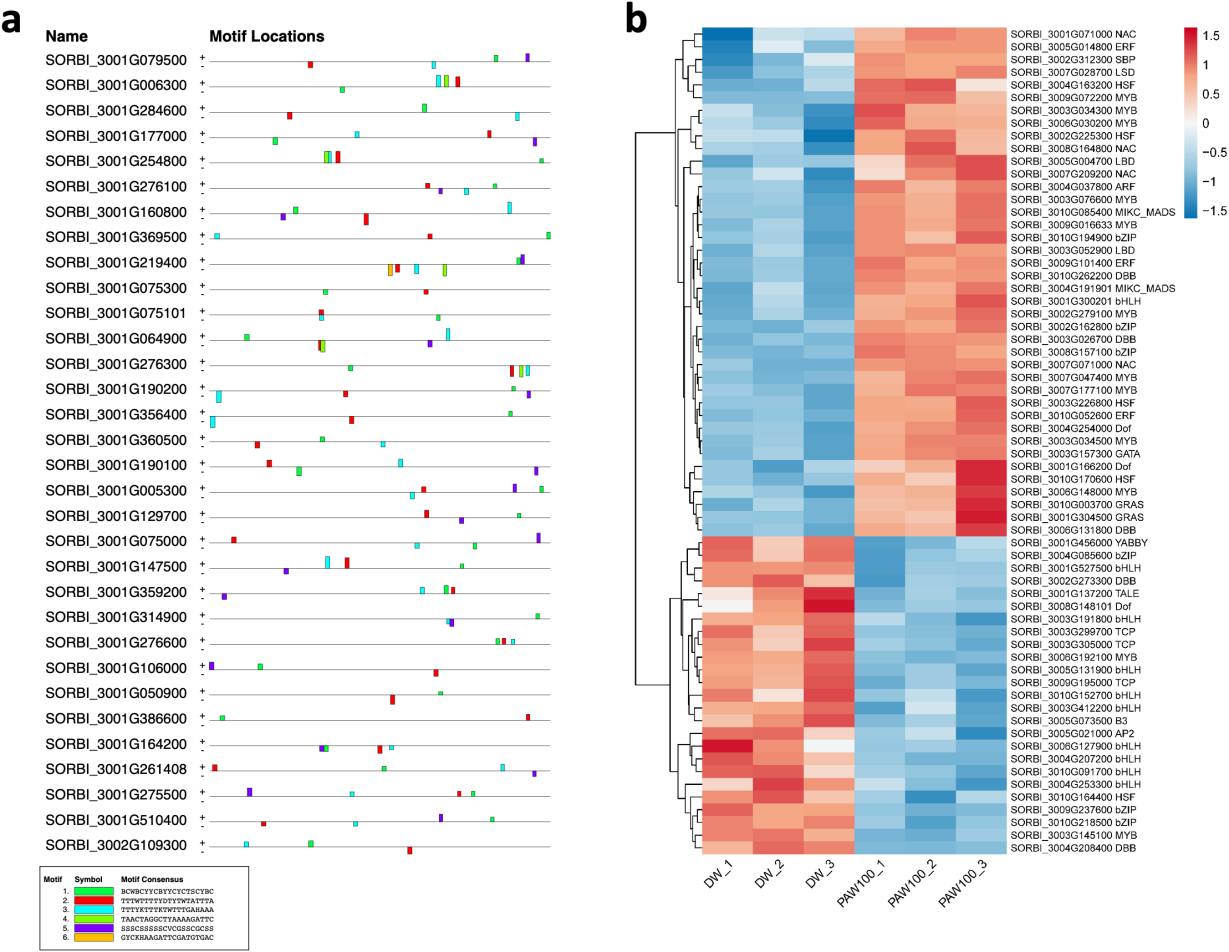
PAW treatment activates TF gene expression. **(A)** Representative binding motifs from isolated GO categories gene. **(B)** Heatmap of TF gene expression in Sorghum under PAW treatment. The heatmap illustrates the dynamic regulation of TF genes, with color gradients representing upregulation (red) and downregulation (blue) across various treatment conditions.

Based on GO analysis, we observed several significantly upregulated GO terms. To further explore the regulatory mechanisms underlying these observations, we performed promoter motif analysis which identified six distinct binding motifs within these promoter regions with varying levels of presence across the gene set (**Figure 6A** and **Table 1**). The most prominent motif, AP2/ERBEP (Motif 1), was detected at 234 sites and matched 56 known motifs from the O’Malley et al. (2016) database. This motif was present in 82.11% of the analyzed genes, suggesting its widespread role in regulating gene expression under the influence of PAW. Another significant motif, C2H2 (motif 2), was found in 224 promoter sites with 75 matches and was present in 78.6% of the genes. Other identified motifs, such as C2C2 (motif 3), AP2/ERBEP (motif 4), G2like (motif 5), and C2C2gata (motif 6), were present in 57.54%, 54.74%, 23.86%, and 10.88% of the genes, respectively, highlighting the diverse regulatory elements that may contribute to the observed highly expressed genes.

**Table 1:** Motif analysis of isolated promoters of highly enriched genes.

No	Conserved motif sequence	Sites	Matches	Top Matched motifs^*^	Percentage
1	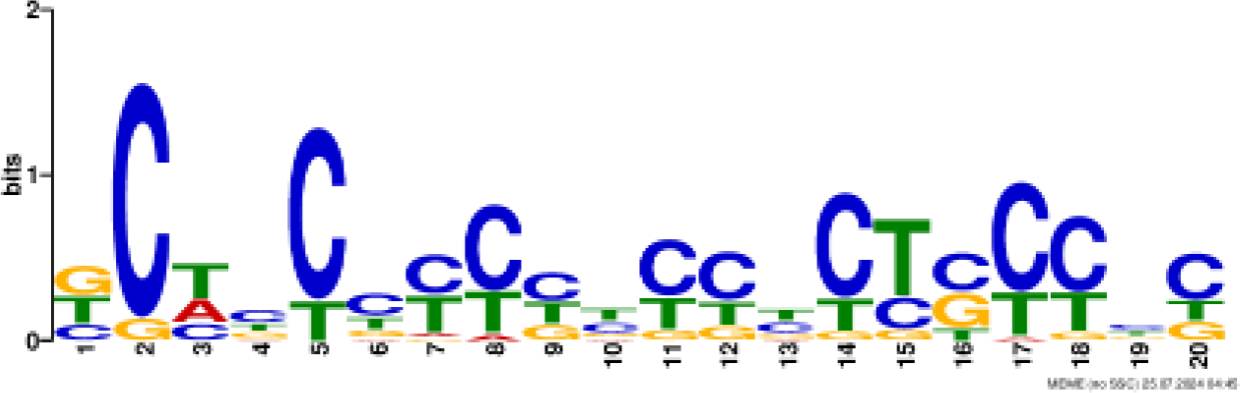	234	56	AP2/ERBEP	82.11%
2	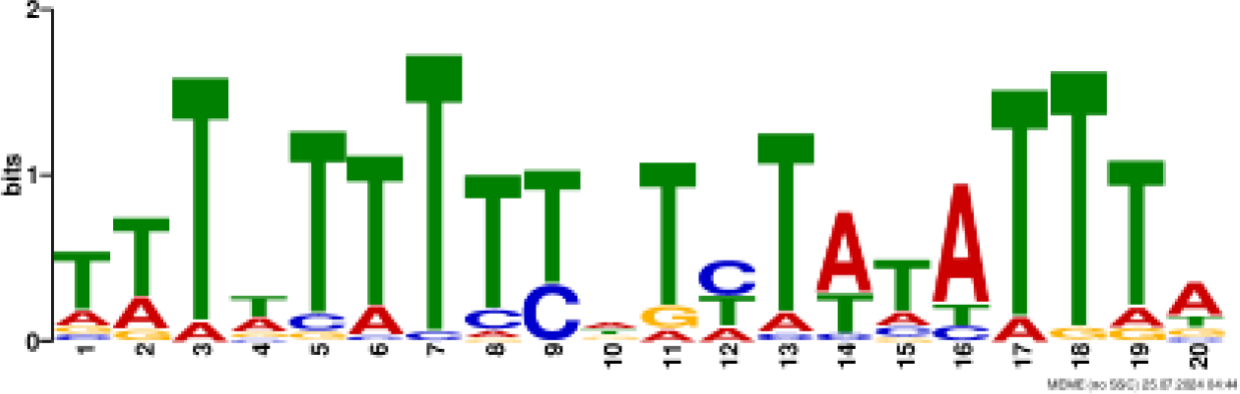	224	75	C2H2	78.6%
3	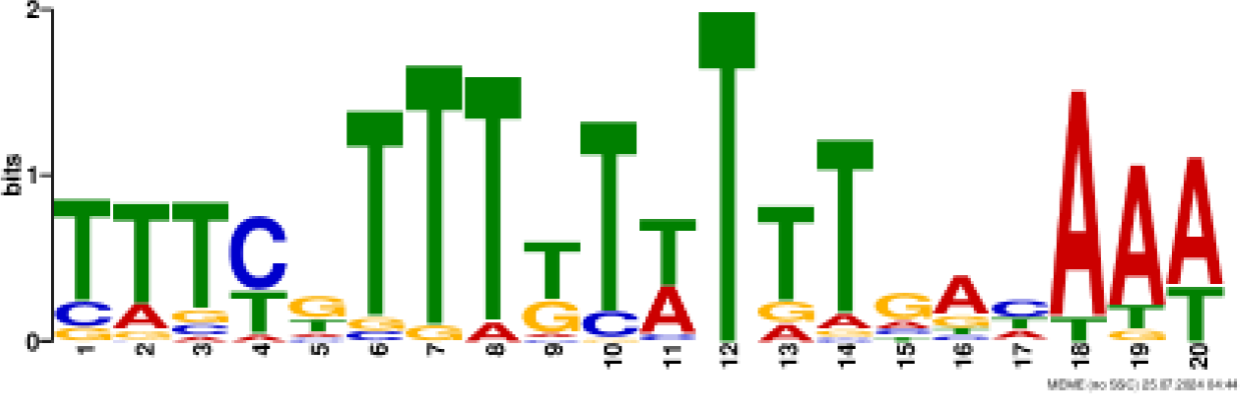	164	56	C2C2	57.54%
4	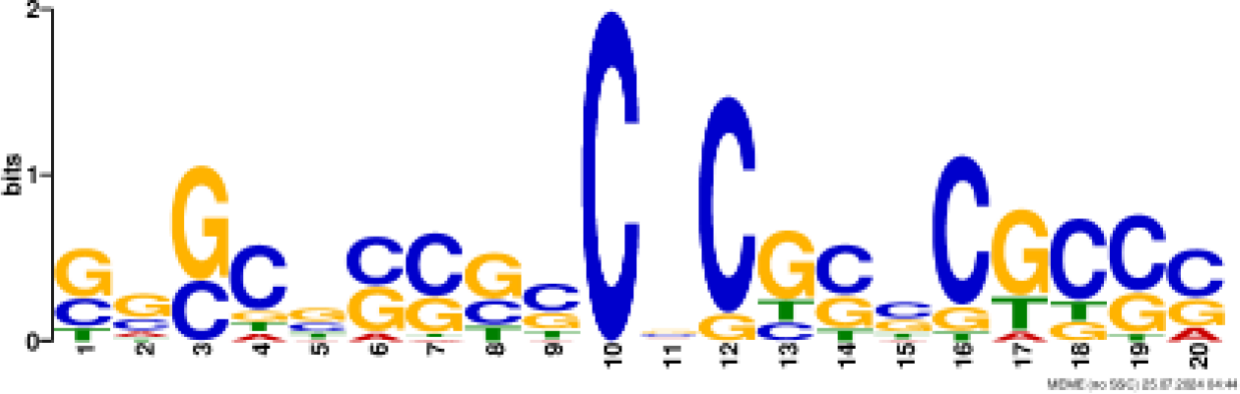	156	77	AP2/EREBP	54.74%
5	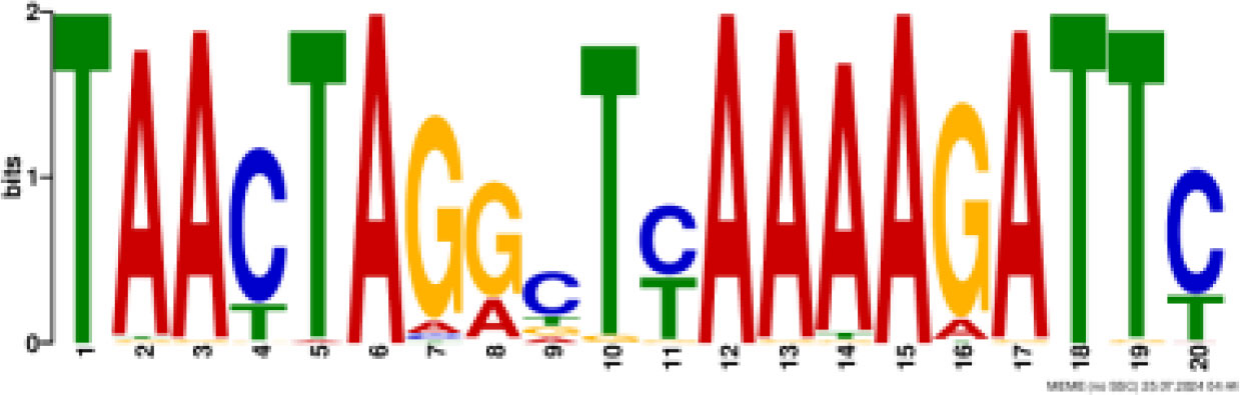	68	15	G2like	23.86%
6	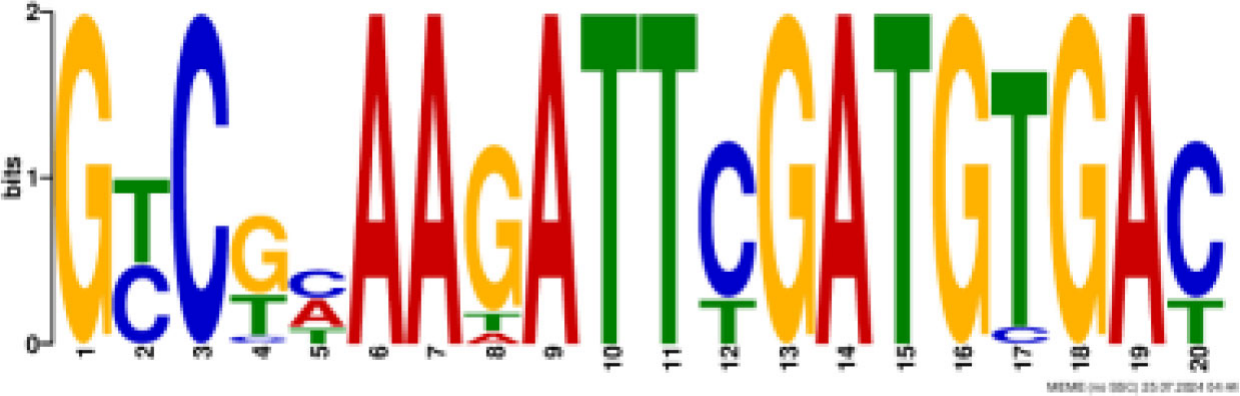	31	10	C2C2gata	10.88%

^*^Based on O’Malley et al. (2016).

To examine the regulatory role of PAW in sorghum gene expression, particularly because of the predominant trend of upregulated genes, we focused on the TF family. TFs are crucial for regulating gene expression in various BPs. Using data from the Plant Transcription Factor Database (https://planttfdb.gao-lab.org/index.php?sp=Sbi), which identified 2,654 TFs (1,859 loci) in sorghum, we analyzed their expression under DW and PAW100 treatments. Our findings revealed that 144 of these TF genes significantly exhibited differential expression, 82% upregulated and 18% down-regulated, in response to PAW treatment compared to the control (**Figure 6B**). Among these, TF families, including 19 Ethylene Response Factors (ERF), 15 basic helix–loop–helix (bHLH), and 14 MYB, were notably differentially expressed in the PAW-treated group.

## 4 Discussion

In this study, we investigated the effects of PAW irrigation on transcriptional regulation during early sorghum growth stages, focusing on the sensitivity of the PAW response of sorghum varieties in terms of plant height, stem thickness, and biomass. Determining the ideal PAW conditions is essential for productive seedling growth, as various sorghum varieties exhibit unique responses to PAW at various concentrations. Our molecular analyses revealed that PAW100 irrigation predominantly activated the expression of genes involved in transmembrane transport, response to light stimuli, mRNA transcription, plasma membrane function, cell wall formation, transcriptional regulatory activity, and oxidoreductase activity. Notably, the AP2/ERBEP motif was highly enriched among the genes upregulated under PAW conditions.

Plants irrigated with PAW promoted growth and biomass, with the PAW100 treatment exhibiting the greatest enhancement. The improved growth after PAW irrigation was likely due to the elevated concentrations of RNS, particularly nitrate (NO_3_^-^), produced by sDBD used in PAW preparation. RNS, such as NO_3_^-^, influences several physiological processes, including seed germination and overall plant growth (Wang et al., 2021). Previous studies using OES have shown that nitrogen-related radicals NO_2_^-^ and NO_3_^-^ are abundant in PAW and are produced when gaseous nitrogen interacts with oxygen atoms, hydroxyl radicals, and water molecules (Lee et al., 2020; Ka et al., 2021). In sorghum, proper PAW irrigation positively impacts plant biomass, whereas longer exposure of plasma to water (PAW400) inhibits sorghum growth owing to excessive NO_3_^-^ concentrations. This phenomenon was also found in other plants; when irrigated with plasma-treated PAW for a long period of time, plant growth was not promoted compared to that in the control group (Adhikari et al., 2019; Lee et al., 2020).

Among the various phenotypic traits examined in the five cultivars and four different PAW conditions, stem thickness was the most significantly affected by PAW treatment in all cultivars. The increase in stem thickness observed in **Figures 2E–G** was closely correlated with the overall increase in biomass, suggesting that stem thickness may be a key indicator of the enhanced growth response under PAW treatment. Given the pronounced differences in stem thickness, we conducted further molecular studies focusing on these tissues to better understand the underlying mechanisms. The cross-section of the basal stem tissue, which contains the SAM and primordia, confirmed the phenotypic differences under PAW irrigation (**Figures 3A–G**). The correlation between stem thickness and biomass accumulation underscores the importance of this parameter in evaluating the effectiveness of PAW treatments on sorghum growth.

GO analysis of the DEGs revealed significant enrichment in BPs essential for plant stress responses and metabolic flexibility. The upregulation of transmembrane transport genes indicates an enhanced capability for nutrient uptake and ion exchange, which are crucial for optimizing metabolic processes essential for growth and development. Several nitrogen and sugar transporter genes such as *NRT1.5* (*NITRATE TRANSPORTER 1.5*), *AMT1;4* (*AMMONIUM TRANSPORTER 1;4*), *SWEET6*, *SWEET12* were highly enriched and distinctly expressed in PAW treatment. These observation aligns with studies in other crop species, where enhanced nutrient transport capabilities have been linked to improved growth outcomes under various stress conditions (Eom et al., 2015; Fan et al., 2021; Pandey et al., 2021). The upregulation of transmembrane transport genes indicates an enhanced capability for nutrient uptake and ion exchange, which are crucial for optimizing metabolic processes essential for growth and development. The increased expression of genes associated with the light response genes such as *CAB1* (*CHLOROPHYLL A/B BINDING PROTEIN 1*), *LHCA4* (*LIGHT-HARVESTING CHLOROPHYLL-PROTEIN COMPLEX I SUBUNIT A4*) likely reflects the effect to the photosynthetic machinery, optimizing energy efficiency and potentially leading to increased biomass accumulation (Friedland et al., 2019; Sekhar et al., 2019; Liu et al., 2023). Furthermore, we observed, enriched genes in GO oxidoreductase activity which have been known to have various function such as oxidative stress response (*TT7*), wax synthesis for defense mechanism (*CER3*), development and catalyze hormone biosynthesis (*KAO2*) (Regnault et al., 2014; Cabello-Hurtado and El Amrani, 2024; Huang et al., 2024). Oxidative stress is a common challenge in plants exposed to abiotic stresses such as drought, salinity, and extreme temperatures, which can severely impede plant growth and productivity. The enhanced expression of these genes in the PAW-treated seedlings implied a greater intrinsic capacity to mitigate oxidative damage, thus maintaining cellular integrity and function during stress exposure.

Our motif analysis revealed that Motif 1, related to the AP2/ERBEP family, was enriched in approximately 82.11% of the upregulated genes, highlighting its potential role in regulatory mechanisms under PAW treatment. The high prevalence of the AP2/ERBEP motif suggests that this TFs family plays a significant role in mediating stress response and developmental regulation in sorghum. The AP2/ERF (APETALA2/Ethylene-Responsive Element Binding Factor) family, often grouped as AP2/ERBEP, is well-known for its involvement in plant stress responses, including drought, salinity, and cold stress (Nakano et al., 2006; Liu et al., 2023). The strong correlation between the AP2/ERBEP motif and the upregulation of transmembrane transport genes suggests that these TFs may enhance the ability of plants to transport nutrients and ions across cellular membranes, which is crucial for maintaining growth and function under stress conditions. This finding aligns with previous studies demonstrating the role of AP2/EREBP TFs in mediating responses to various environmental stimuli (Vidal et al., 2010; Singh et al., 2022). The high enrichment of the AP2/EREBP motifs suggests that PAW may enhance the transcriptional activity of genes by recognizing stress inducers involved in nitrate uptake and metabolism, thereby promoting plant growth.

Because the majority of DEG were upregulated, we further examined TF gene expression. Upon examining the transcriptional levels of TF genes, it was discovered that 82% of TFs had an up regulated during PAW irrigation. In particular, TFs that regulate transcription showed increased expression when sorghum was exposed to PAW, indicating a positive regulatory role for sorghum stem DEGs. Several TF families known to play important roles in growth, biomass accumulation, and stress responses were significantly affected, suggesting that PAW may modulate key regulatory pathways that control plant development and adaptability to environmental stress.

Among the affected TF families, ERF, bHLH, MYB, and NAC were the most notably enriched. These families are known for their roles in plant growth, development, and stress response. For instance, AP2/ERF are well known for their involvement in mediating responses to environmental stresses, such as drought, salinity, and cold (Liu et al., 2023; Ma et al., 2024). MYB TFs are involved in the regulation of secondary metabolism, cell-fate determination, and responses to biotic and abiotic stressors (Dubos et al., 2010; Yang et al., 2022). Similarly, NAC TFs play critical roles in cell division, expansion, and stress signaling (Olsen et al., 2005; Nuruzzaman et al., 2013). The enrichment of these TF families in response to PAW suggests that PAW may enhance these fundamental processes, thereby contributing to improved growth and stress resilience.

Plasma generates RONS in water, including nitrates, and is an effective treatment for enhancing nutrient availability and uptake. Nitrate is a crucial macronutrient for plant development and influences processes such as seed germination, root and leaf growth, and overall yield. Previous studies have shown that nitrate availability can directly affect the expression of nitrate-responsive genes, many of which are regulated by AP2/EREBP TF (Li et al., 2022b). This supports our hypothesis that the PAW-induced upregulation of these TFs is a key mechanism driving the observed biomass increase in sorghum seedlings. Moreover, the broader impact of PAW on various TF families indicates that PAW treatment may trigger a network of regulatory pathways, enhancing not only growth, but also the ability of the plant to withstand environmental stresses. This response underscores the potential of PAW as a sustainable agricultural practice, providing a viable alternative to chemical fertilizers or priming agents for seedlings.

Although numerous case studies have been conducted on the enhancement of plant biomass and growth by PAW, few tissue-specific findings have been observed in plants. Here, we found that PAW treatment altered the expression of a substantial number of genes, including TF, through conserved key regulatory motifs, such as AP2/EREBP, thereby modulating crucial biological pathways in sorghum. These physiological and molecular findings provide strong evidence for the potential of PAW as a growth-promoting alternative in agriculture, primarily through the induction of stress- and development-related pathways. PAW irrigation has the potential to significantly boost agricultural productivity by modulating transcriptional activation, and consequently improving early plant development.

Investigating the effects of PAW irrigation on crop yield and quality is important for field application of PAW in sustainable agriculture. Future studies on the molecular mechanisms that enhance harvesting and boost natural product production in a range of plant species should explore the potential benefits of PAW irrigation. This research contributes to improving the application of eco-friendly PAW technology to crops, ensuring environmental sustainability by applying it to agriculture, and increasing agricultural production.

## Data Availability

The original contributions presented in the study are publicly available. The names of the repository/repositories and accession number(s) can be found in the article/**Supplementary Material.**
