# Supplementary material for: Biomass enhancement and activation of transcriptional regulation in sorghum seedling by plasma-activated water
Source: Front Plant Sci. 2024 Nov 22;15:1488583. doi: 10.3389/fpls.2024.1488583 (PMC11620867; doi:10.3389/fpls.2024.1488583)
Supplement: Supplementary file 1 [file DataSheet1.docx]

Supplementary Material

**Biomass Enhancement and Activation of Transcriptional Regulation in Sorghum Seedling by Plasma-Activated Water**

Hong Kwan Beak^1, 2 ,†^ , Ryza A. Priatama ^1,,†^, Sang-Ik Han^3^, Soon Ju Park^4^ and Young Koung Lee^1,5*^

^1^Institute of Plasma Technology, Korea Institute of Fusion Energy, Gunsan 54004, Republic of Korea

^2^Division of Biological Sciences, Wonkwang University, Iksan 54538, Republic of Korea

^3^ Upland Crop Breeding Research Division Department of Southern Area Crop Science. National Institute of Crop Science, Rural Development Administration, Miryang 50424, Republic of Korea

^4^ Division of Applied Life Science and Plant Molecular Biology and Biotechnology Research Center (PMBBRC), Gyeongsang National University, Jinju, 52828, Republic of Korea

^5^ Plasma and Nuclear Fusion, University of Science and Technology, Daejeon 34113, Republic of Korea

^†^ These authors contributed equally to this work.

*** Correspondence:**Corresponding Author : Young Koung Lee
leeyk@kfe.re.kr

**
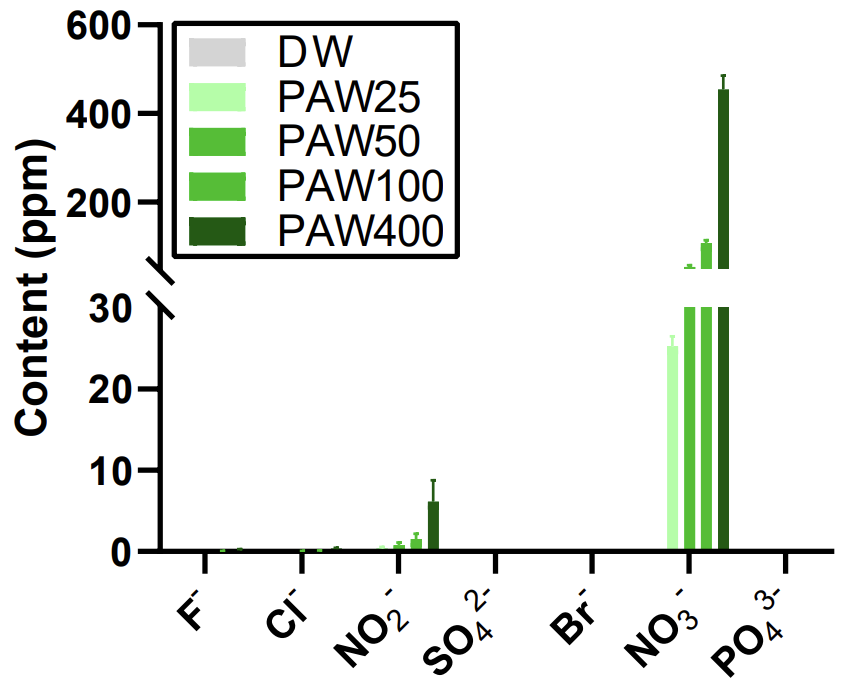
**

# Figure S1. Ion chromatography (IC) analysis of anion content at different PAW concentrations.

# The concentrations of various anions are shown for Fluoride (F⁻), Chloride (Cl⁻), Nitrite (NO₂⁻), Sulfate (SO₄²⁻), Bromide (Br⁻), Nitrate (NO₃⁻), and Phosphate (PO₄³⁻) across PAW treatments (DW, PAW25, PAW50, PAW100, and PAW400). The y-axis represents anion content in parts per million (ppm), with error bars showing standard deviations from five replicate measurements.

**
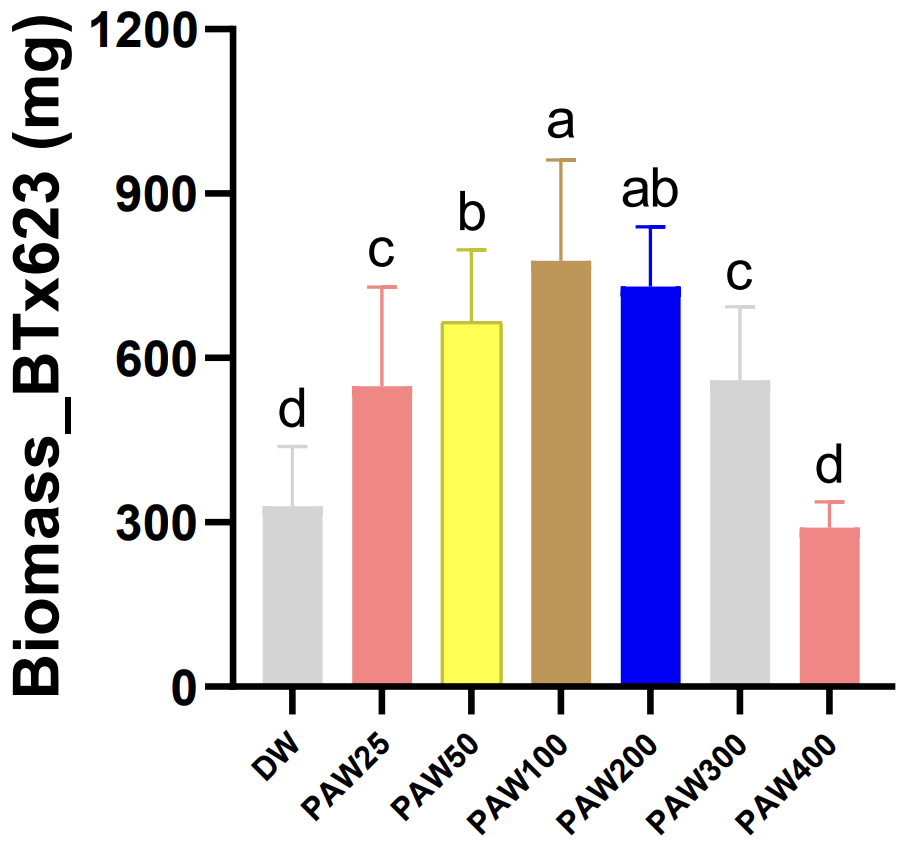
**

**Figure S2. Effect of Plasma Activated Water (PAW) concentration on the biomass of sorghum (BTx623).**

Bar plots represent the mean biomass (in mg) ± standard deviation (SD) for each treatment group. Different letters above the bars indicate statistically significant differences between groups (ANOVA, Tukey’s HSD, *P* ≤ 0.05). Sorghum seedlings were grown in soil for 20 days, and biomass measurements were recorded at the end of this period.

**Figure S3. PAW treatment-induced sorghum leaf phenotype.**

(a) Leaf area. (b) Leaf length. (c) Leaf width. (d) Fully expanded leaf number. (e) Chlorophyll content. Bar plots represent mean ± standard deviation (SD) and different letters indicate statistically significant differences (ANOVA, Tukey's HSD, *P* ≤ 0.05). Seedlings were grown in soil for 20 days. All phenotypes were recorded 20 days after sowing.

#

**Figure S4. Read counts and volcano plot of differentially expressed genes (DEG) analysis.**

(a) Total raw read counts from RNA-seq samples, showing the number of reads (in millions) for each replicate in the DW (control) and PAW100 treatments. (b) Volcano plot showing DEGs between DW and PAW100 treatments. Genes meeting the threshold of 1.5-fold change (p-value < 0.05, FDR < 0.1) are highlighted. Upregulated genes are shown in red, downregulated genes in green, and non-significant genes are represented in gray.

**
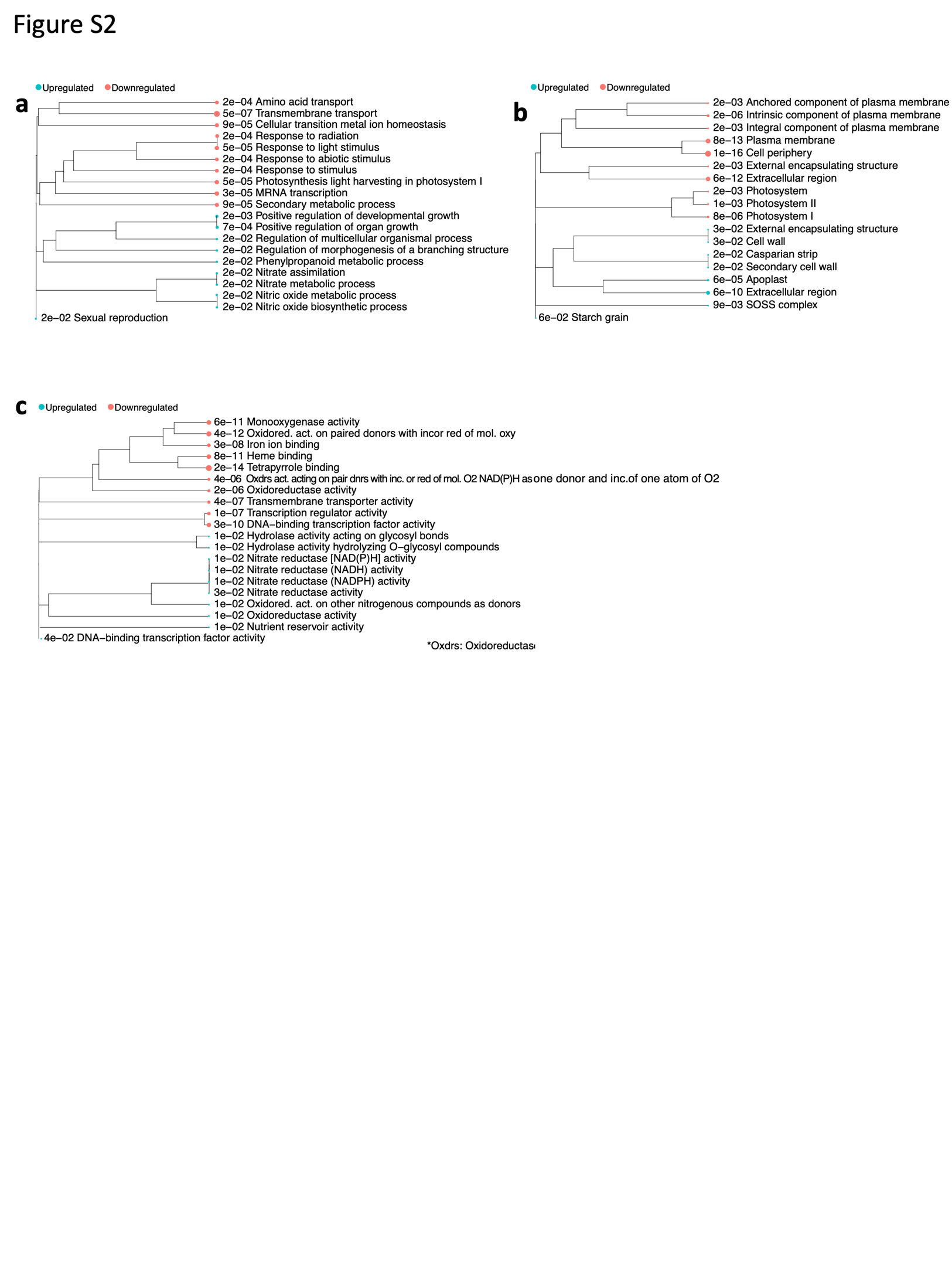
**

**Figure S5. Tree map of enriched Gene Ontology (GO) analysis of FC1.5 differentially expressed genes.**

GO categories are shown for (a) Biological process, highlighting major pathways such as response to stimulus, photosynthesis, and nitrate metabolism; (b) Cellular component, including plasma membrane, cell wall, and photosystem regions; and (c) Molecular function, focusing on oxidoreductase activity, ion binding, and nitrate reductase activity. Upregulated genes are marked in blue, while downregulated genes are marked in red.

**Figure S6. Gene Ontology (GO) analysis of differentially expressed genes (DEGs) in cellular component and molecular function categories.**

(a) Bar plot of enriched cellular components, with the size of the dots representing the number of genes associated with each GO term, and the color gradient indicating fold enrichment. (b) Bar plot of enriched molecular functions, showing the distribution of DEGs across categories such as oxidoreductase and transcription regulator activity. (c) GO-network of cellular components, illustrating interactions between terms where the size of nodes corresponds to the number of genes and the color gradient reflects enrichment levels. (d) GO-network of molecular functions, depicting connections between highly enriched terms. All analyses are based on DEGs with a fold change threshold of 1.5, p-value < 0.05, and FDR < 0.1.

**Supplementary Table 1. Summary of successfully mapped reads from RNA-seq analysis.**

| Sample | Total reads | Mapped reads | Mapping rate (%) | Count(>0) | Exp(>1) |
| --- | --- | --- | --- | --- | --- |
| DW-1 | 34,184,054 | 31,811,635 | 93.06 | 26,956 | 19,961 |
| DW-2 | 29,325,114 | 27,228,838 | 92.85 | 26,777 | 20,053 |
| DW-3 | 24,245,016 | 22,490,699 | 92.76 | 26,236 | 19,838 |
| PAW100-1 | 33,609,593 | 29,996,688 | 89.25 | 27,008 | 20,344 |
| PAW100-2 | 27,999,942 | 25,621,473 | 91.51 | 26,751 | 20,148 |
| PAW100-3 | 27,225,731 | 25,298,746 | 92.92 | 26,878 | 20,276 |

DW (Distilled water as control) and PAW100 (Plasma-activated water 100) treatment groups.
